# Supplementary material for: A simeprevir-inducible molecular switch for the control of cell and gene therapies
Source: Nat Commun. 2023 Nov 27;14:7753. doi: 10.1038/s41467-023-43484-9 (PMC10682029; doi:10.1038/s41467-023-43484-9)

Supplementary Information

A simeprevir-inducible molecular switch for the control of cell and gene therapies

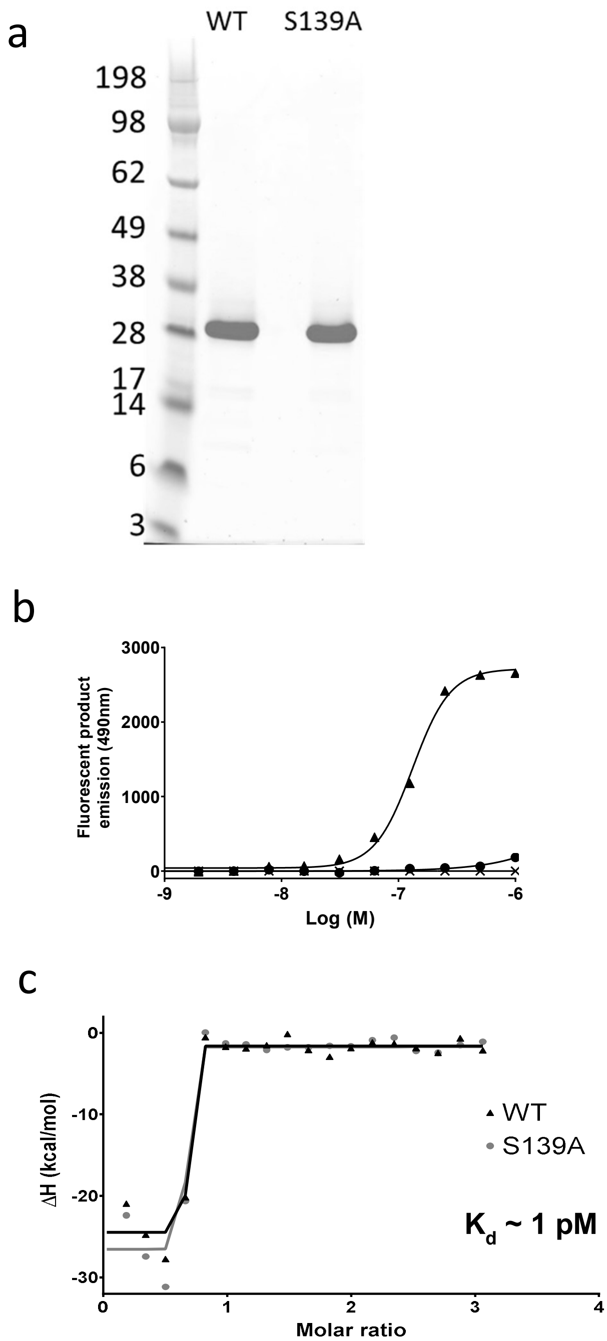

Supplementary Fig 1: Characterization of the S139A active site variant of HCV NS3/4A PR. (a) SDS-PAGE gel of purified recombinant wild type (WT) and S139A HCV NS3/4A PR. (b) S139A mutant of HCV NS3/4A PR exhibits minimal activity relative to WT HCV NS3/4A PR in a peptide cleavage assay. To assess enzyme activity, cleavage of a fluorogenic HCV protease FRET substrate with an EDANS-Dabcyl donor-quencher pair (RET S1, AnaSpec) was measured. When in close proximity (10–100 Å), energy emitted from EDANS will be quenched by Dabcyl. Cleavage releases EDANS, allowing detection of fluorescence at 490nm. Serial dilutions of HCV protease NS3/NS4A and the active site mutant S139A in assay buffer (HEPES pH 7.8, 5mM DTT, 100mM NaCl, 10% glycerol, 0.01% CHAPS) were incubated with fluorogenic substrate at room temperature. Fluorescence was measured after 3 hours using a PerkinElmer Envision plate reader (excitation 340nm, emission 490nm). Each data point represents the mean of two independent experiments. Dose response curves were fit to the data using 4 parameter nonlinear regression. (c) The WT and S139A version of

HCV NS3/4A PR exhibit equivalent affinity as measured by isothermal calorimetry. Isothermal calorimetry (ITC) was carried out using the Auto-ITC200 (Malvern), with a preliminary injection of 0.4 µl followed by 19 injections of 2 µl each, at 120 second intervals. Rotation of the solution was set to 750 rpm and temperature 37°C. Simeprevir (125 µM) was titrated into HCV NS3/4A PR (WT 8 µM and S139A mutant 8.2 µM) or protein buffer (control); the protein buffer was enriched with 2.5% DMSO to equal the amount present in the simeprevir solution. The WT was run once. The S139A mutant was run twice, giving similar results both times; a representative experiment is shown. The data were analysed with the ITC-PEAQ software (Malvern) using a one-site binding model and reference subtraction point-by-point. Source data are provided as a Source Data file.

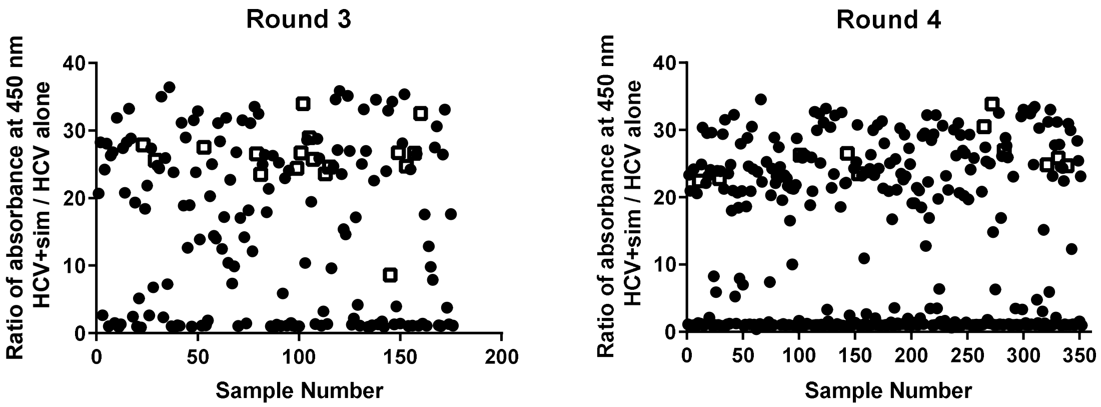

Supplementary Fig 2: Selectivity of individual clones from phage display selection outputs 3 and 4 for binding to the HCV NS3/4A PR:simeprevir (sim) complex over the HCV NS3/4A PR alone was determined by phage ELISA. Squares represent the Tn3 clones (see Supplementary Table 1) that were chosen for further characterization. Source data are provided as a Source Data file.

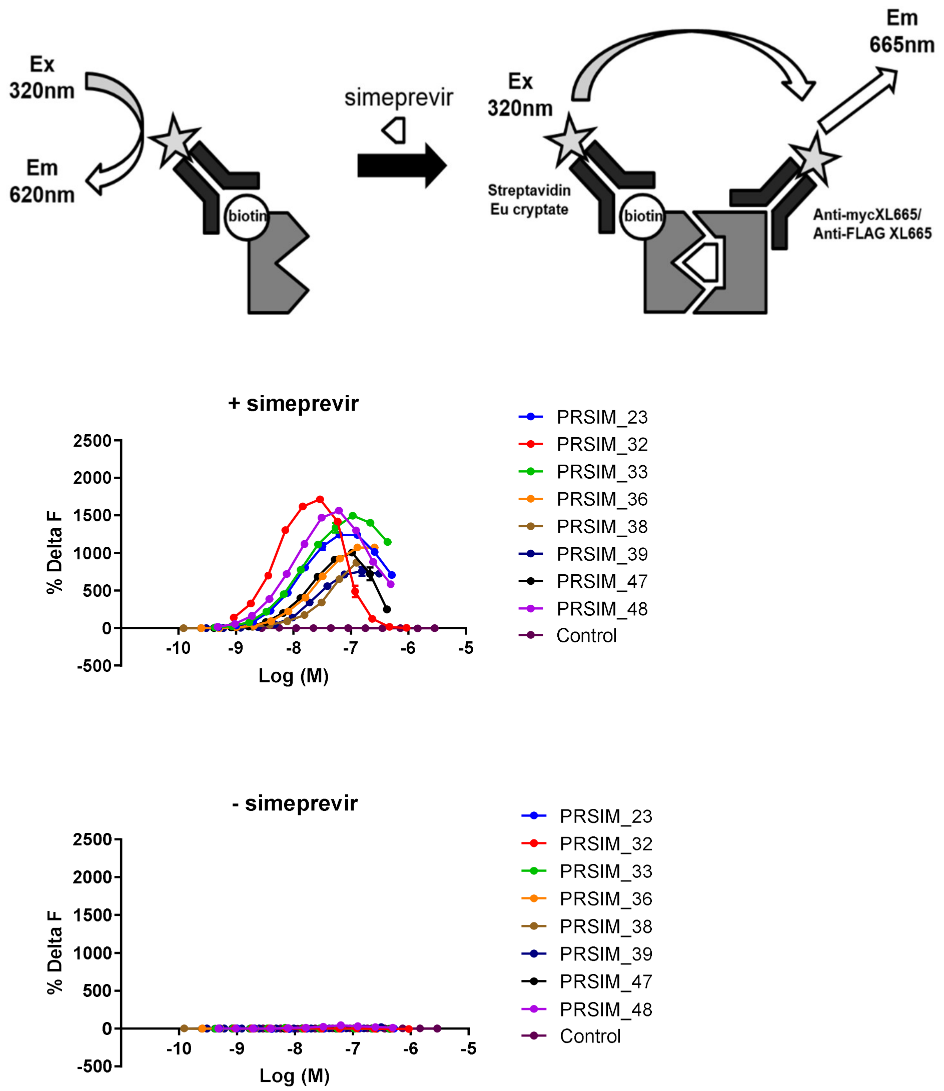

Supplementary Fig 3: (Top) Schematic of the homogeneous time-resolved fluorescence (HTRF) assay employed to measure the binding of PRSIM molecules to HCV NS3/4A PR (S139A) alone or in complex with simeprevir. Ex is excitation; Em is emission. (Middle) PRSIM molecules binding to the HCV NS3/4A PR:simeprevir complex but not the HCV NS3/4A PR alone (bottom) in an HTRF assay. Each data point represents the mean of two independent experiments. Source data are provided as a Source Data file.

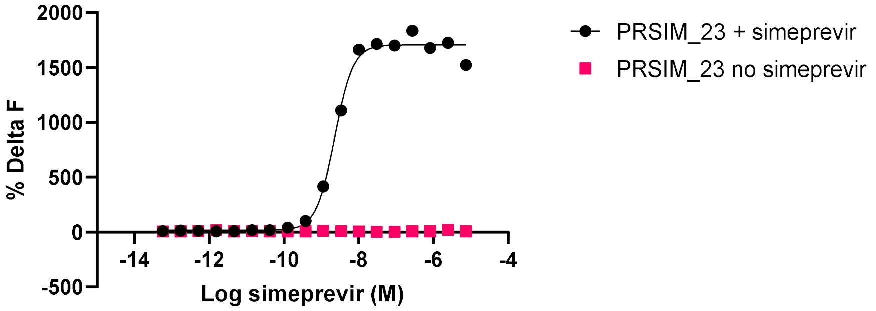

Supplementary Fig. 4: The ability of free simeprevir in solution to compete with the binding of HCV PR-simeprevir complex to PRISM_23 was measured using the HTRF assay shown in Supplementary Fig. 3. The concentrations of HCV_PR and PRISM_23 were fixed at 5 nM and 6 nM, respectively, and complex formation was measured in the presence of a titration of simeprevir ranging from 57 fM to 7.4 µM. No inhibition of complex formation is observed a high concentrations of simeprevir, indicating that simeprevir does not interact with PRSIM_23 alone at these concentrations. Each data point represents the mean of two independent experiments. Dose response curves were fit to the data using 4 parameter nonlinear regression. Source data are provided as a Source Data file.

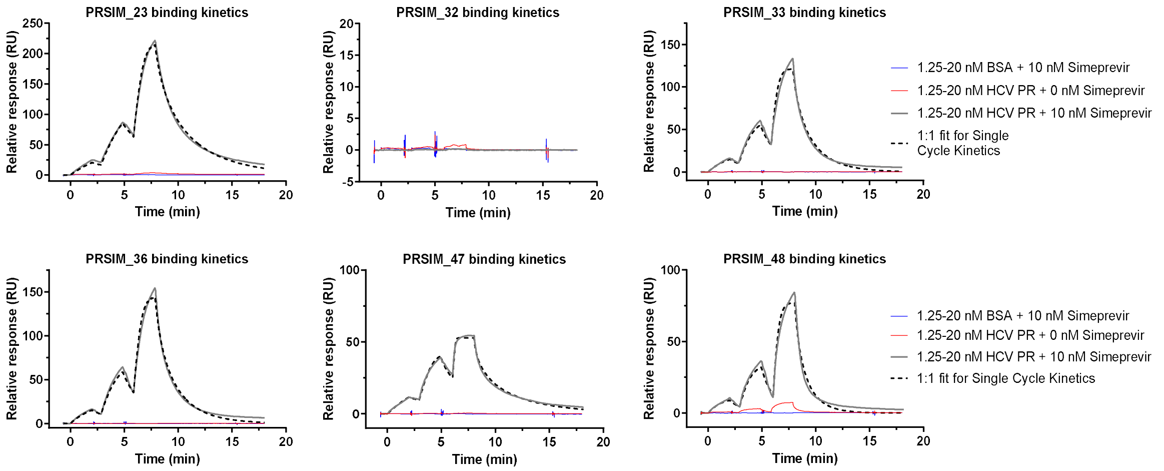

Supplementary Fig. 5: Binding of HCV PR, or BSA control, to PRSIM binding molecules in the presence or absence of simeprevir. The interactions between HCV PR (or BSA) and PRSIM binding molecules were measured by Surface Plasmon Resonance (SPR) using a Biacore 8K instrument at 25 °C. HCV PR bound to five PRSIM binding molecules strongly and selectively in the presence of simeprevir (grey lines). Only one molecule (PRSIM_48) showed significant non-specific binding to HCV PR alone (red lines). BSA did not bind to the PRSIM binding molecules in the presence of simeprevir (blue lines). Solid grey lines represent measured data points and the dashed black lines represent the 1:1 fit for Single Cycle Kinetics data used for analysis. Source data are provided as a Source Data file.

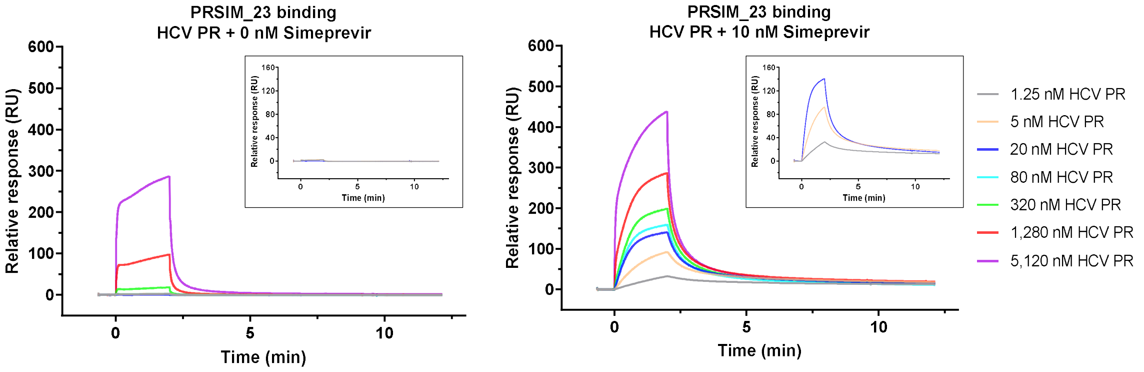

Supplementary Fig. 6: Binding of HCV PR to PRSIM_23 is very sensitive in the presence of low concentration (10 nM) of simeprevir. The interactions between HCV PR and PRSIM_23 were measured by Surface Plasmon Resonance (SPR) using a Biacore 8K instrument at 25 °C. The HCV PR was diluted 1:4 (1.25-5,120 nM) ± 10 nM simeprevir in 10 mM HEPES pH 7.4, 150 mM NaCl, 0.05% Surfactant P20, 0.01% DMSO. PRSIM_23 was immobilized onto CM5 chips and the samples were flowed over the chip at 50 µl/min using multi cycle kinetics, with 2 minute association and 10 minute dissociation. (Left) In the absence of simeprevir the HCV PR shows low affinity binding to PRSIM_23. However, there is also an element of non-specific binding, demonstrated by the shape of the curves. (Right) In the presence of 10 nM simeprevir the binding signal is increased and clear binding is seen even at the lowest HCV PR concentrations (inserts). Source data are provided as a Source Data file.

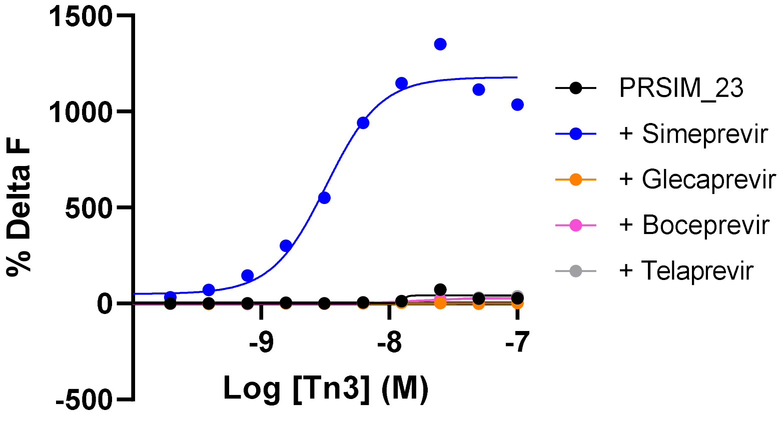

Supplementary Fig 7: PRSIM_23 binds to HCV PR in complex with simeprevir (blue closed circles) but not to HCV PR in complex with covalent HCV protease inhibitors (orange, pink, grey) in an HTRF assay. Black circles indicate complex formation in the absence of simeprevir. Each data point represents the mean of two independent experiments. Dose response curves were fit to the data using 4 parameter nonlinear regression. Source data are provided as a Source Data file.

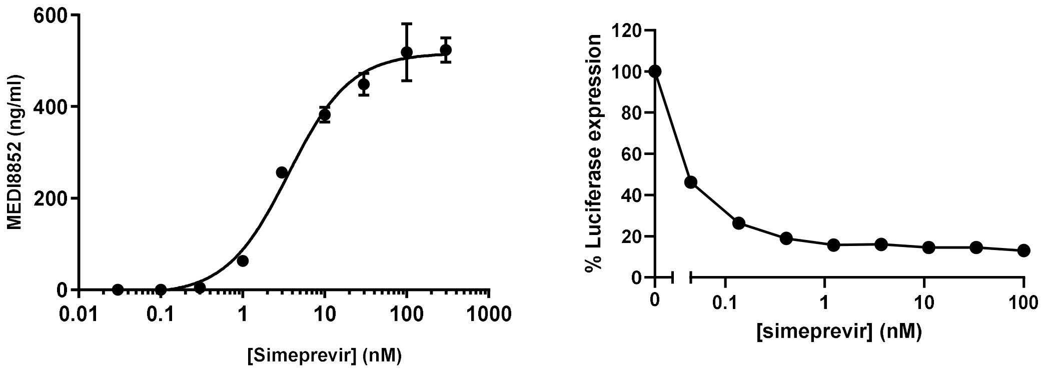

Supplementary Fig. 8: (Left) Simeprevir-induced dose-dependent expression of MEDI8852 IgG via the PRSIM_23-CID module-based split transcription factor. MEDI8852 concentrations were measured using the Singleplex Human/NHP IgG Isotyping Kit (Mesoscale Discovery). Each data point represents the mean ± s.e.m of six independent experiments. Dose response curves were fit to the data using 4 parameter nonlinear regression. (Right) Simeprevir dose-dependent inhibition of luciferase expression after induction of shRNA targeting luciferase via the PRSIM_23-CID module-based split transcription factor. Luminescence observed at each concentration of simeprevir was normalized to the luminescence observed in the absence of simeprevir. Each data point represents the mean ± s.e.m of three independent experiments. Source data are provided as a Source Data file.

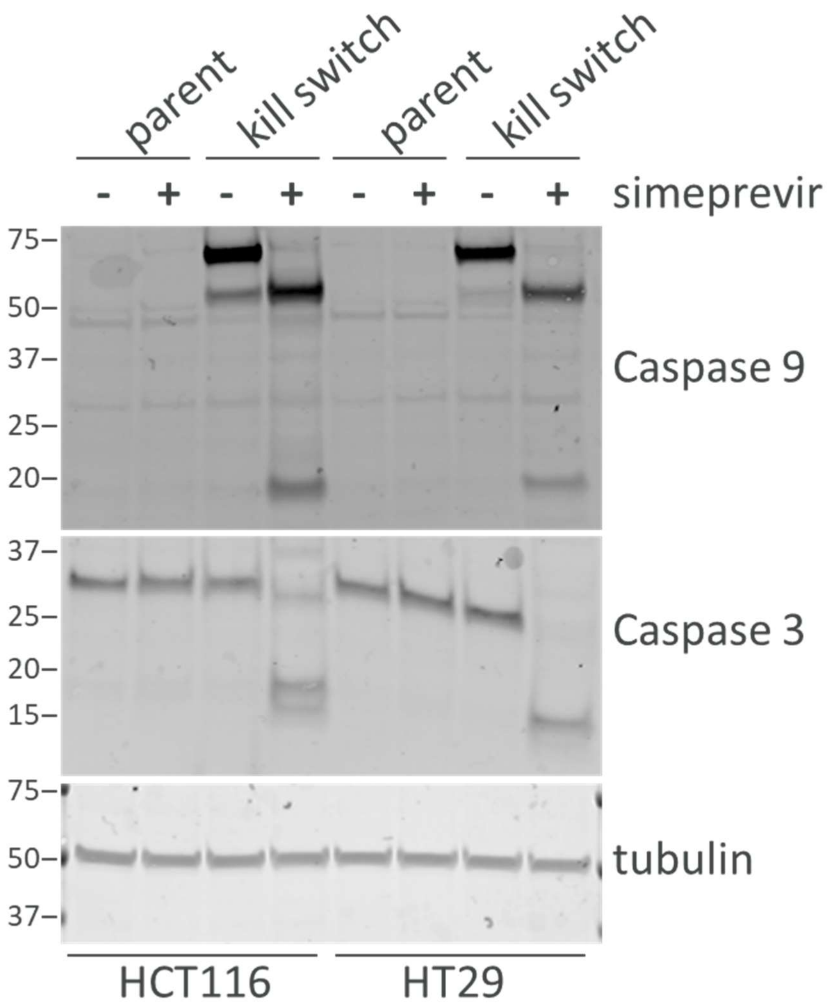

Supplementary Fig. 9: Western blot of cell lysates shows cleavage of Caspase9 incorporated into the PRSIM_23-based kill switch and downstream Caspase 3 in HCT116 and HT29 cells stably transduced with the kill switch, but not in the parental cells after 3-4h treatment with 10nM simeprevir. Tubulin is the loading control. Antibodies used were from Cell Signalling Technology: 9502S (Caspase 9), 9662S (Caspase 3) and 3837S (tubulin), at dilutions of 1:1500, 1:1500 and 1:2000, respectively. A representative of n=2 independent experiments is shown.

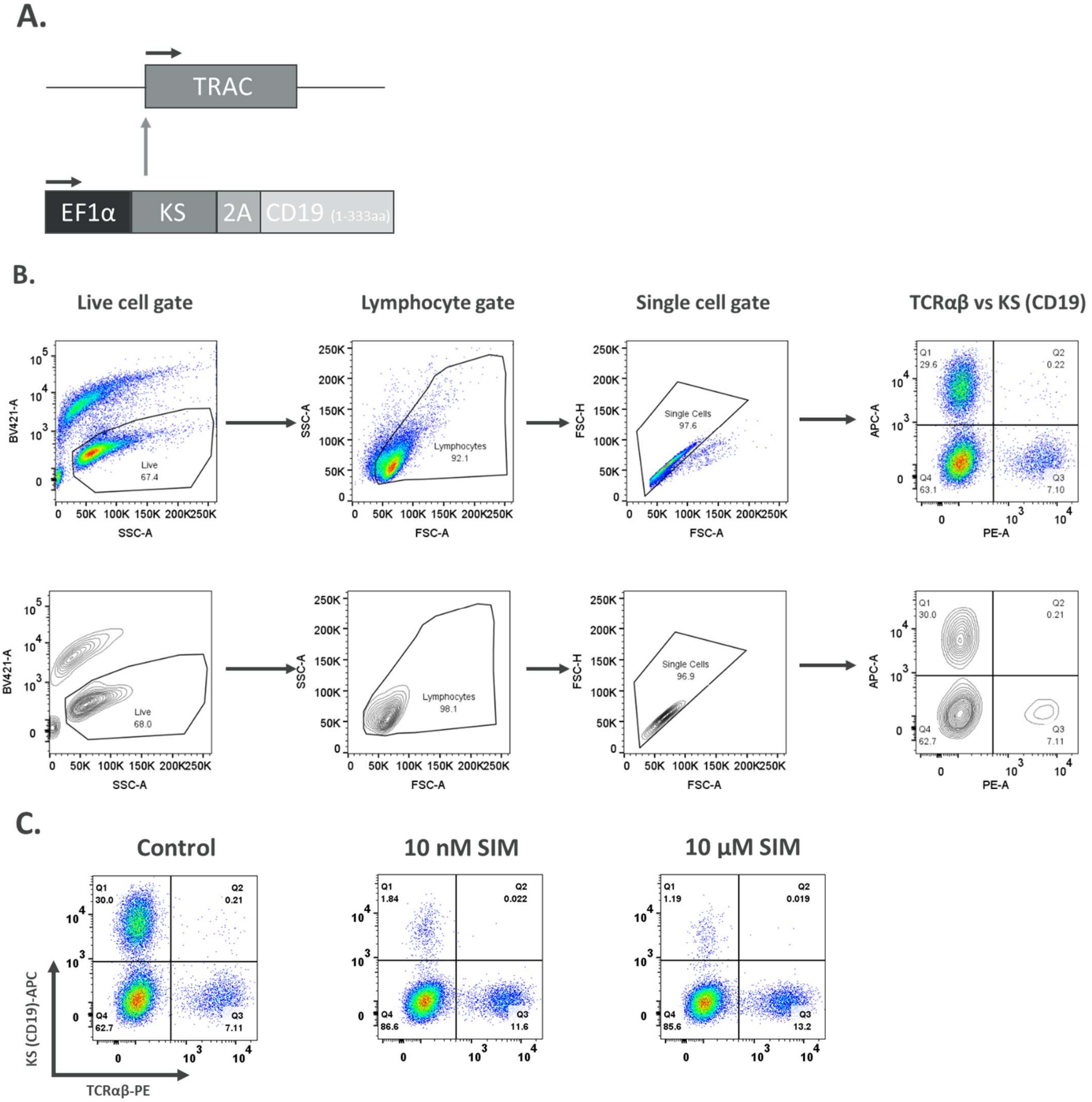

Supplementary Fig. 10: A. Primary human CD8+ T Lymphocytes were engineered via a CRISPR/ Cas9 Knock-Out (KO)/ Knock-In (KI) strategy to express a minimal EF1α promoter driven Kill switch (KS) -2A-GPI anchored CD19 (1-33 amino acids) cassette in place of the endogenous T cell receptor (TCR). B. Gating strategy used to identify percentages of cell populations relevant to this analysis: (1) Live cell gate (2) Lymphocyte gate (3) Doublet exclusion gate (4) % KS-CD19+ TCRαβ- (TRAC/iCasp9 KI cells). C. Functionality of the PRSIM-based kill switch was determined by flow cytometry, measuring the disappearance of live KS+ (CD19+) cells following Simeprevir (SIM; 10 nM or 10 µM) treatment for 7, 14 or 21 days. Representative flow cytometry data for one experiment is shown.

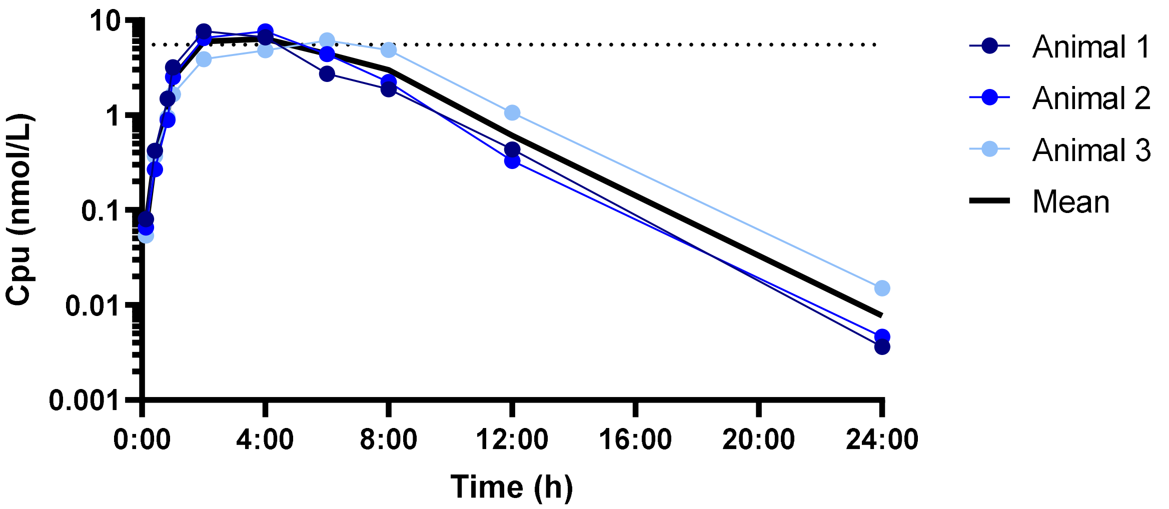

Supplementary Fig. 11: Pharmacokinetics of simeprevir (5% dimethyl sulfoxide, 95% sulfobutylether-β-cyclodextrin (30% w/v) in water) dosed orally at 200 mg/kg in female Balb/c mice. This study was carried out at Pharmaron. Unbound plasma concentration (Cpu) was calculated from the measuring total plasma concentration using a fraction unbound value for simeprevir in mouse plasma of 0.0009. The dashed line indicates the measured in vitro EC50 for cell killing for kill-switch transduced HT29 cells after 4 h of 5.5 nM. Blue circles represent the data for each individual mouse and the solid black line is the mean of the measurements for the three mice. Source data are provided as a Source Data file.

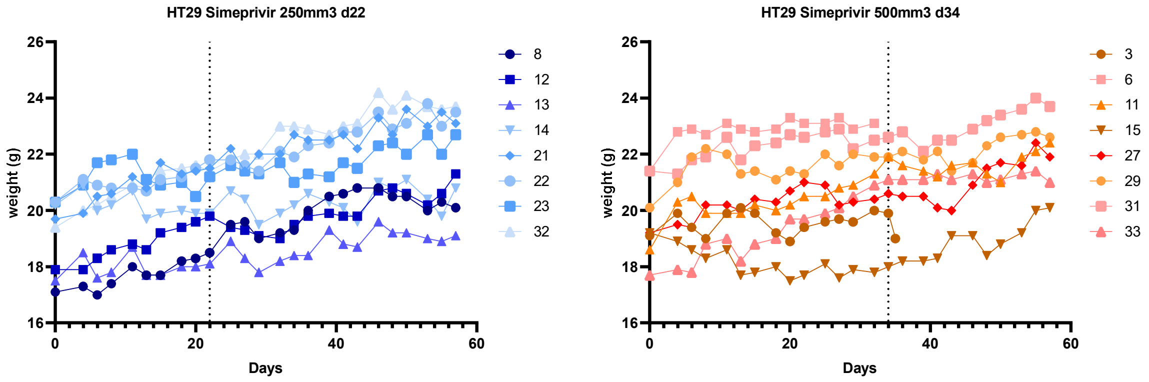

Supplementary Fig. 12: Body weight measurements of individual mice in the kill-switch transduced HT29 xenograft mouse model. The dashed line represents the day of simeprevir dosing. Each line represents an individual mouse. Blue symbols are mice treated with simeprevir after average group tumor size reached 250 mm^3 and orange symbols are mice treated with simeprevir after average group tumor size reached 500 mm^3. Source data are provided as a Source Data file.

Supplementary Tables

Supplementary Table 1: Tn3 PRSIM binding molecules that are selective for the HCV PR:simeprevir complex over HCV PR alone were identified in homogeneous time-resolved fluorescence (HTRF) assays run in parallel to measure binding in the presence and absence of simeprevir.

Clone Name	Selection round	Binding fold change [(HCV protease + simeprevir binding) / HCV protease alone]	HTRF max. signal (% delta F)
**PRSIM_23**	**4**	**23.8**	**1573**
PRSIM_24	3	31.5	561
PRSIM_25	4	24.0	577
PRSIM_26	4	25.5	304
PRSIM_27	4	25.3	422
PRSIM_28	3	27.1	692
PRSIM_29	3	26.0	365
PRSIM_30	3	25.4	550
PRSIM_31	4	25.0	351
**PRSIM_32**	**4**	**22.4**	**1955**
**PRSIM_33**	**3**	**29.9**	**1704**
PRSIM_34	3	22.2	614
PRSIM_35	3	24.8	437
**PRSIM_36**	**3**	**27.9**	**1440**
PRSIM_37	3	25.3	867
**PRSIM_38**	**3**	**23.3**	**1061**
**PRSIM_39**	**4**	**24.9**	**1015**
PRSIM_40	3	26.1	218
PRSIM_41	4	22.8	964
PRSIM_42	3	8.8	1895
PRSIM_43	3	28.6	317
PRSIM_44	3	25.6	340
PRSIM_45	4	33.3	842
PRSIM_46	3	33.3	362
**PRSIM_47**	**3**	**25.3**	**1367**
**PRSIM_48**	**3**	**26.6**	**1780**
PRSIM_49	4	30.0	761
PRSIM_50	4	26.3	897

Supplementary Table 2: Kinetics of HCV PR binding to PRSIM binding molecules in the presence of simeprevir.

		HCV NS3/NS4A (S139A) + 10 nM simeprevir			
	**ID**	**k_a (M^{-1} s^{-1})**	**k_d (s^{-1})**	**K_D (nM)**	**%R_{max}¥ (RUs)**
**Tn3**	PRSIM_23	6.07 (± 1) × 10^6	3.72 (± 0.5) × 10^{-2}	6.14 (± 0.2)	24 (± 4)
	PRSIM_32	N.D.	N.D.	#	N.D.
	PRSIM_33	*6.71 (± 9) × 10^8*	*3.88 (± 5) × 10^0*	*6.99 (± 1.4)*	*12 (± 3)*
	PRSIM_36	8.67 (± 3) × 10^6	9.63 (± 4) × 10^{-2}	10.9 (± 1)	19 (± 1)
	PRSIM_47	4.16 (± 5) × 10^{10}	7.90 (± 9) × 10^1	1.96 (± 0.1)	23 (± 1)
	PRSIM_48	*4.24 (± 7) × 10^9*	*4.04 (± 7) × 10^1*	*12.2 (± 2.4)*	*15 (± 5)*

The interactions between HCV PR and PRSIM binding molecules were measured by Surface Plasmon Resonance (SPR) using a Biacore 8K instrument at 25 °C. The PRSIM binding molecules were immobilized onto CM5 chips. The HCV PR in the presence of simeprevir was then flowed over the chips in sequentially increasing concentrations (single cycle kinetics). The equilibrium dissociation constant (K_D) was determined using a 1:1 binding model. Data are presented as mean ± standard deviation from 3 independent experiments. N.D. = indicates the values could not be determined due to absence of detectable binding. # = no binding. Data in italics indicates high association rate and lower than expected R_{max}. ¥= % of theoretical R_{max}.

Supplementary Table 3: Kinetics of HCV PR binding to PRSIM_23 in the absence of simeprevir.

	**HCV NS3/NS4A (S139A) + 0 nM Simeprevir**			
**Tn3**	**k_a (M^{-1} s^{-1})**	**k_d (s^{-1})**	**K_D (µM)**	**%R_{max} ¥ (RUs)**
**PRSIM_23**	1.50 (± 0.04) × 10^4	9.22 (± 0.5) × 10^{-2}	6.2 (± 0.4)	233 (± 10)

The interaction between HCV PR and PRSIM_23 was measured by Surface Plasmon Resonance (SPR) using a Biacore 8K instrument at 25 °C. The PRSIM_23 was immobilized onto a CM5 chip. The HCV PR in the absence of simeprevir was then flowed over the chips in sequentially increasing concentrations (single cycle kinetics). The equilibrium dissociation constant (K_D) was determined using a 1:1 binding model. Data are presented as average ± standard deviation from 4 independent experiments. ¥= % of theoretical R_{max}.

Supplementary Table 4: Protein and guide RNA constructs used in this study.

Description	protein /DNA	Sequence
6His-Avi-HCV NS3/4A PR	protein	MGSSHHHHHHGSGLNDIFEAQKIEWHEGGGGSMKKKGSVVIVGRINLSGDTAYAQQTRGEEGCQETSQTG<br>RDKNQVEGEVQIVSTATQTFLATSINGVLWTVYHGAGTRTIASPKGPVTQMYTNVDKDLVGWQAPQGSRSLT<br>PCTCGSSDLYLVTRHADVIPVRRRGDSRGSLLSPRPISYLKGSSGGPLLCPAGHAVGIFRAAVSTRGVAKAVD<br>FIPVESLETTMRSP
6His-Avi-HCV NS3/4A PR (S139A)	protein	MGSSHHHHHHGSGLNDIFEAQKIEWHEGGGGSMKKKGSVVIVGRINLSGDTAYAQQTRGEEGCQETSQTG<br>RDKNQVEGEVQIVSTATQTFLATSINGVLWTVYHGAGTRTIASPKGPVTQMYTNVDKDLVGWQAPQGSRSLT<br>PCTCGSSDLYLVTRHADVIPVRRRGDSRGSLLSPRPISYLKGSAGGPLLCPAGHAVGIFRAAVSTRGVAKAVD<br>FIPVESLETTMRSP
PRSIM_23	protein	RLDAPSQIEVKDVTDTTALITWVDPRYDDIWWFELTYGIKDVPGDRTTIKLYLNDPYYSIGNLKPDTEYEVSLIS<br>YTGDSYSRSGSNPAKITFKTGL
PRSIM_32	protein	RLDAPSQIEVKDVTDTTALITWWSPRYYYASISGFELTYGIKDVPGDRTTIKLDYASNDYSIGNLKPDTEYEVSLI<br>SWNYGDWRYSSSNPAKITFKTGL
PRSIM_33	protein	RLDAPSQIEVKDVTDTTALITWYPPGRWYDDIWYFELTYGIKDVPGDRTTIKLARGDDVYSIGNLKPDTEYEVS<br>LISWGPDRGDRAGSNPAKITFKTGL
PRSIM_36	protein	RLDAPSQIEVKDVTDTTALITWSWPRDDDYDIWYFELTYGIKDVPGDRTTIKLLNYASPYSIGNLKPDTEYEVSL<br>ISVVPDTYGRGTSNPAKITFKTGL
PRSIM_38	protein	RLDAPSQIEVNVTDTTALITWDTPGDDVDNIWYFELTYGIKDVPGDRTTIKLSAGDGDYSIGNLKPDTEYEVSLI<br>SVGPGTVTRDGSNPAKITFKTGL
PRSIM_39	protein	RLDAPSQIEVKDVTDTTALITWRSPDDDDYNIWYFELTYGIKDVPGDRTTIKLAYDDDYYSIGNLKPDTEYEVSL<br>ISVGDRPWYYYRSNPAKITFKTGL
PRISM_47	protein	RLDAPSQIEVKDVTDTTALITWSRPGVSIWYFELTYGIKDVPGDRTTIKLDYRSYYYSIGNLKPDTEYEVSLISGS<br>YGLVGVRASNPAKITFKTGL
PRISM_48	protein	RLDAPSQIEVKDVTDTTALITWRLPVLGIYRFELTYGIKDVPGDRTTIKLWSSPSNYSIGNLKPDTEYEVSLISGR<br>PYDSSNPAKITFKTGL
HCV PR-p65 AD	protein	MGKKKGSVVIVGRINLSGDTAYAQQTRGEEGCQETSQTGRDKNQVEGEVQIVSTATQTFLATSINGVLWTVY<br>HGAGTRTIASPKGPVTQMYTNVDKDLVGWQAPQGSRSLTPCTCGSSDLYLVTRHADVIPVRRRGDSRGSLL<br>SPRPISYLKGSAGGPLLCPAGHAVGIFRAAVSTRGVAKAVDFIPVESLETTMRSP
ZFHD1 DBD-PRSIM_23	protein	MDYPAAKRVKLDSRERPYACPVESCDRRFSRSDELTRHIRIHTGQKPFQCRICMRNFSRSDHLTTHIRTHTGG<br>GRRRKKRTSIETNIRVALEKSFLENQKPTSEEITMIADQLNMEKEVIRVWFCNRRQKEKRINTSAGSRLDAPSQ<br>IEVKDVTDTTALITWVDPRYDDIWWFELTYGIKDVPGDRTTIKLYLNDPYYSIGNLKPDTEYEVSLISYTGDSYS<br>RSGSNPAKITFKTGL
ZFHD1 DBD-(PRSIM_23)$_2$	protein	MDYPAAKRVKLDSRERPYACPVESCDRRFSRSDELTRHIRIHTGQKPFQCRICMRNFSRSDHLTTHIRTHTGG<br>GRRRKKRTSIETNIRVALEKSFLENQKPTSEEITMIADQLNMEKEVIRVWFCNRRQKEKRINTSAGSRLDAPSQ<br>IEVKDVTDTTALITWVDPRYDDIWWFELTYGIKDVPGDRTTIKLYLNDPYYSIGNLKPDTEYEVSLISYTGDSYS<br>RSGSNPAKITFKTGLRLDAPSQIEVKDVTDTTALITWVDPRYDDIWWFELTYGIKDVPGDRTTIKLYLNDPYYSI<br>GNLKPDTEYEVSLISYTGDSYSRSGSNPAKITFKTGL
ZFHD1 DBD-(PRSIM_23)$_3$	protein	MDYPAAKRVKLDSRERPYACPVESCDRRFSRSDELTRHIRIHTGQKPFQCRICMRNFSRSDHLTTHIRTHTGG<br>GRRRKKRTSIETNIRVALEKSFLENQKPTSEEITMIADQLNMEKEVIRVWFCNRRQKEKRINTSAGSRLDAPSQ<br>IEVKDVTDTTALITWVDPRYDDIWWFELTYGIKDVPGDRTTIKLYLNDPYYSIGNLKPDTEYEVSLISYTGDSYS<br>RSGSNPAKITFKTGLRLDAPSQIEVKDVTDTTALITWVDPRYDDIWWFELTYGIKDVPGDRTTIKLYLNDPYYSI<br>GNLKPDTEYEVSLISYTGDSYSRSGSNPAKITFKTGLRLDAPSQIEVKDVTDTTALITWVDPRYDDIWWFELTY<br>GIKDVPGDRTTIKLYLNDPYYSIGNLKPDTEYEVSLISYTGDSYSRSGSNPAKITFKTGL
DBD-(PRSIM_23)$_3$-P2A-HCV PR-p65 AD	protein	MDYPAAKRVKLDSRERPYACPVESCDRRFSRSDELTRHIRIHTGQKPFQCRICMRNFSRSDHLTTHIRTHTGG<br>GRRRKKRTSIETNIRVALEKSFLENQKPTSEEITMIADQLNMEKEVIRVWFCNRRQKEKRINTSAGSRLDAPSQ<br>IEVKDVTDTTALITWVDPRYDDIWWFELTYGIKDVPGDRTTIKLYLNDPYYSIGNLKPDTEYEVSLISYTGDSYS<br>RSGSNPAKITFKTGLRLDAPSQIEVKDVTDTTALITWVDPRYDDIWWFELTYGIKDVPGDRTTIKLYLNDPYYSI<br>GNLKPDTEYEVSLISYTGDSYSRSGSNPAKITFKTGLRLDAPSQIEVKDVTDTTALITWVDPRYDDIWWFELTY<br>GIKDVPGDRTTIKLYLNDPYYSIGNLKPDTEYEVSLISYTGDSYSRSGSNPAKITFKTGLTSKGSGATNFSLLKQ<br>AGDVEENPGPMAKKGSVVIVGRINLSGDTAYAQQTRGEEGCQETSQTGRDKNQVEGEVQIVSTATQTFLATS<br>INGVLWTVYHGAGTRTIASPKGPVTQMYTNVDKDLVGWQAPQGSRSLTPCTCGSSDLYLVTRHADVIPVRRR<br>GDSRGSLLSPRPISYLKGSAGGPLLCPAGHAVGIFRAAVSTRGVAKAVDFIPVESLETTMRSPTRDEFPTMVF

		PSGQISQASALAPAPPQVLPQAPAPAPAPAMVSALAQAPAPVPVLAPGPPQAVAPPAPKPTQAGEGTLSEAL<br>LQLQFDDEDLGALLGNSTDPAVFTDLASVDNSEFQQLLNQGIPVAPHTTEPMLMEYPEAITRLVTGAQRPPDP<br>APAPLGAPGLPNGLLSGDEDFSSIADMDFSALLSQISSTSY
dCas9-<br>(PRSIM_23)$_3$	protein	MDYYPYDVPDYADKKYSIGLAIGTNSVGWAVITDEYKVPSKKFKVLGNTDRHSIKKNLIGALLFDSGETAEATR<br>LKRTARRRYTRRKNRICYLQEIFSNEMAKVDDSFFHRLEESFLVEEDKKHERHPIFGNIVDEVAYHEKYPTIYHL<br>RKKLVDSTDKADLRLIYLALAHMIKFRGHFLIEGDLNPDNSDVDKLFIQLVQTYNQLFEENPINASGVDAKAILSA<br>RLSKSRRLENLIAQLPGEKKNGLFGNLIALSLGLTPNFKSNFDLAEDAKLQLSKDTYDDDLDNLLAQIGDQYAD<br>LFLAAKNLSDAILLSDILRVNTEITKAPLSASMIKRYDEHHQDLTLLKALVRQQLPEKYKEIFFDQSKNGYAGYID<br>GGASQEEFYKFIKPILEKMDGTEELLVKLNREDLLRKQRTFDNGSIPHQIHLGELHAILRRQEDFYPFLKDNREK<br>IEKILTFRIPYYVGPLARGNSRFAWMTRKSEETITPWNFEEVVDKGASAQSFIERMTNFDKNLPNEKVLPKHSL<br>LYEYFTVYNELTKVKYVTEGMRKPAFLSGEQKKAIVDLLFKTNRKVTVKQLKEDYFKKIECFDSVEISGVEDRF<br>NASLGTYHDLLKIIKDKDFLDNEENEDILEDIVLTLTLFEDREMIEERLKTYAHLFDDKVMKQLKRRRYTGWGRL<br>SRKLINGIRDKQSGKTILDFLKSDGFANRNFMQLIHDDSLTFKEDIQKAQVSGQGDSLHEHIANLAGSPAIKKGI<br>LQTVKVVDELVKVMGRHKPENIVIEMARENQTTQKGQKNSRERMKRIEEGIKELGSQILKEHPVENTQLQNEK<br>LYLYYLQNGRDMYVDQELDINRLSDYDVDAIVPQSFLKDDSIDNKVLTRSDKNRGKSDNVPSEEVVKKMKNY<br>WRQLLNAKLITQRKFDNLTKAERGGLSELDKAGFIKRQLVETRQITKHVAQILDSRMNTKYDENDKLIREVKVIT<br>LKSKLVSDFRKDFQFYKVREINNYHHAHDAYLNAVVGTALIKKYPKLESEFVYGDYKVYDVRKMIAKSEQEIGK<br>ATAKYFFYSNIMNFFKTEITLANGEIRKRPLIETNGETGEIVWDKGRDFATVRKVLSMPQVNIVKKTEVQTGGFS<br>KESILPKRNSDKLIARKKDWDPKKYGGFDSPTVAYSVLVVAKVEKGKSKKLKSVKELLGITIMERSSFEKNPIDF<br>LEAKGYKEVKKDLIIKLPKYSLFELENGRKRMLASAGELQKGNELALPSKYVNFLYLASHYEKLKGSPEDNEQK<br>QLFVEQHKHYLDEIIEQISEFSKRVILADANLDKVLSAYNKHRDKPIREQAENIIHLFTLTNLGAPAAFKYFDTTID<br>RKRYTSTKEVLDATLIHQSITGLYETRIDLSQLGGDPKKKRKVGSAGSRLDAPSQIEVKDVTDTTALITWVDPR<br>YDDIWWFELTYGIKDVPGDRTTIKLYLNDPYYSIGNLKPDTEYEVSLISYTGDSYSRSGSNPAKITFKTGLRLDA<br>PSQIEVKDVTDTTALITWVDPRYDDIWWFELTYGIKDVPGDRTTIKLYLNDPYYSIGNLKPDTEYEVSLISYTGD<br>SYSRSGSNPAKITFKTGLRLDAPSQIEVKDVTDTTALITWVDPRYDDIWWFELTYGIKDVPGDRTTIKLYLNDPY<br>YSIGNLKPDTEYEVSLISYTGDSYSRSGSNPAKITFKTGL.
HCV PR - VPR<br>AD	protein	MGKKKGSVVIVGRINLSGDTAYAQQTRGEEGCQETSQTGRDKNQVEGEVQIVSTATQTFLATSINGVLWTVY<br>HGAGTRTIASPKGPVTQMYTNVDKDLVGWQAPQGSRSLTPCTCGSSDLYLVTRHADVIPVRRRGDSRGSLL<br>SPRPISYLKGSAGGPLLCPAGHAVGIFRAAVSTRGVAKAVDFIPVESLETTMRSPTGGGGSGGGGSEASGSG<br>RADALDDFDLDMLGSDALDDFDLDMLGSDALDDFDLDMLGSDALDDFDLDMLINSRSSGSPKKKRKVGSQYL<br>PDTDDRHRIEEKRKRTYETFKSIMKKSPFSGPTDPRPPPRRIAVPSRSSASVPKPAPQPYPFTSSLSTINYDEF<br>PTMVFPSGQISQASALAPAPPQVLPQAPAPAPAPAMVSALAQAPAPVPVLAPGPPQAVAPPAPKPTQAGEGT<br>LSEALLQLQFDDEDLGALLGNSTDPAVFTDLASVDNSEFQQLLNQGIPVAPHTTEPMLMEYPEAITRLVTGAQ<br>RPPDPAPAPLGAPGLPNGLLSGDEDFSSIADMDFSALLGSGSGSRDSREGMFLPKPEAGSAISDVFEGREVC<br>QPKRIRPFHPPGSPWANRPLPASLAPTPTGPVHEPVGSLTPAPVPQPLDPAPAVTPEASHLLEDPDEETSQA<br>VKALREMADTVIPQKEEAAICGQMDLSHPPPRGHLDELTTTLESMTEDLNLDSPLTPELNEILDTFLNDECLLH<br>AMHISTGLSIFDTSLF
PRSIM_23-HCV<br>PR-Casp9<br>activation<br>domain	protein	MGSRLDAPSQIEVKDVTDTTALITWVDPRYDDIWWFELTYGIKDVPGDRTTIKLYLNDPYYSIGNLKPDTEYEV<br>SLISYTGDSYSRSGSNPAKITFKTGLGGGSGMKKKGSVVIVGRINLSGDTAYAQQTRGEEGCQETSQTGRDK<br>NQVEGEVQIVSTATQTFLATSINGVLWTVYHGAGTRTIASPKGPVTQMYTNVDKDLVGWQAPQGSRSLTPCT<br>CGSSDLYLVTRHADVIPVRRRGDSRGSLLSPRPISYLKGSAGGPLLCPAGHAVGIFRAAVSTRGVAKAVDFIPV<br>ESLETTMRSPGGGSGVDGFGDVGALESLRGNADLAYILSMEPCGHCLIINNVNFCRESGLRTRTGSNIDCEKL<br>RRRFSSLHFMVEVKGDLTAKKMVLALLELARQDHGALDCCVVVILSHGCQASHLQFPGAVYGTDGCPVSVEK<br>IVNIFNGTSCPSLGGKPKLFFIQACGGEQKDHGFEVASTSPEDESPGSNPEPDATPFQEGLRTFDQLDAISSLP<br>TPSDIFVSYSTFPGFVSWRDPKSGSWYVETLDDIFEQWAHSEDLQSLLLRVANAVSVKGIYKQMPGCFNFLR<br>KKLFFKTS
PRSIM_23-HCV<br>PR-Casp9<br>activation<br>domain (S196A)	protein	MGSRLDAPSQIEVKDVTDTTALITWVDPRYDDIWWFELTYGIKDVPGDRTTIKLYLNDPYYSIGNLKPDTEYEV<br>SLISYTGDSYSRSGSNPAKITFKTGLGGGSGMKKKGSVVIVGRINLSGDTAYAQQTRGEEGCQETSQTGRDK<br>NQVEGEVQIVSTATQTFLATSINGVLWTVYHGAGTRTIASPKGPVTQMYTNVDKDLVGWQAPQGSRSLTPCT<br>CGSSDLYLVTRHADVIPVRRRGDSRGSLLSPRPISYLKGSAGGPLLCPAGHAVGIFRAAVSTRGVAKAVDFIPV<br>ESLETTMRSPGGGSGVDGFGDVGALESLRGNADLAYILSMEPCGHCLIINNVNFCRESGLRTRTGSNIDCEKL<br>RRRFSALHFMVEVKGDLTAKKMVLALLELARQDHGALDCCVVVILSHGCQASHLQFPGAVYGTDGCPVSVEK<br>IVNIFNGTSCPSLGGKPKLFFIQACGGEQKDHGFEVASTSPEDESPGSNPEPDATPFQEGLRTFDQLDAISSLP<br>TPSDIFVSYSTFPGFVSWRDPKSGSWYVETLDDIFEQWAHSEDLQSLLLRVANAVSVKGIYKQMPGCFNFLR<br>KKLFFKTS

Suppl. Fig 1b: Uncropped SDS-PAGE gel

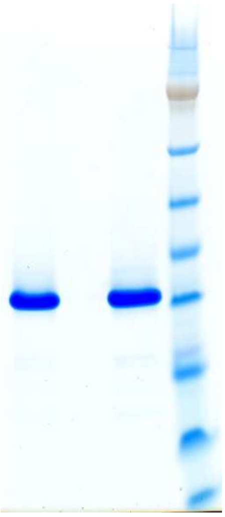

Suppl. Fig 9: Uncropped Western blots

Caspase 9:

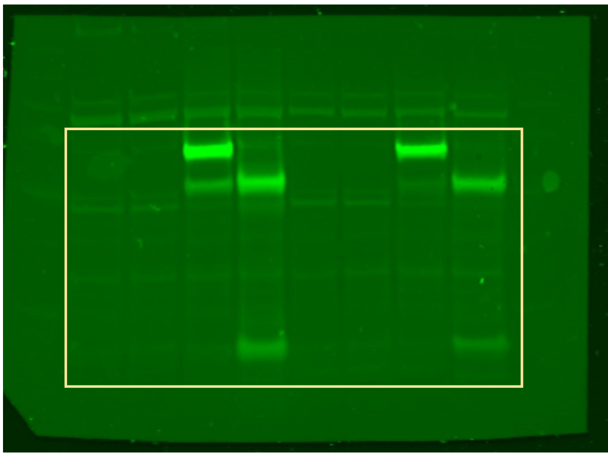

Caspase 3:

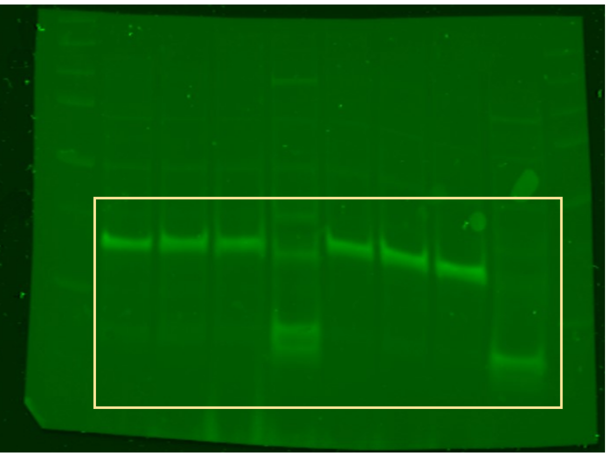

Tubulin:

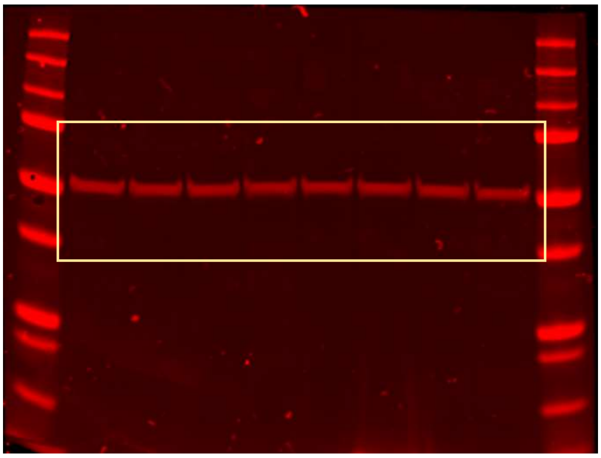

Supplement: Supplementary file 1 — Supplementary Information [file 41467_2023_43484_MOESM1_ESM.pdf]
